# Impacts of the COVID‐19 pandemic on vegetable production systems and livelihoods: Smallholder farmer experiences in Burkina Faso

**DOI:** 10.1002/fes3.337

**Published:** 2021-10-31

**Authors:** B. Jan Middendorf, Hamidou Traoré, Gerad Middendorf, Prakash K. Jha, Djibril Yonli, Siébou Palé, P.V. Vara Prasad

**Affiliations:** ^1^ Feed the Future Innovation Lab for Collaborative Research on Sustainable Intensification Kansas State University Manhattan Kansas USA; ^2^ Institut de l’Environnement et de Recherches Agricoles (INERA) Ouagadougou Burkina Faso; ^3^ Department of Sociology, Anthropology, and Social Work Kansas State University Manhattan Kansas USA; ^4^ Department of Agronomy Kansas State University Manhattan Kansas USA

**Keywords:** Burkina Faso, COVID‐19, resilience, smallholder farmers, vegetable production systems

## Abstract

At the onset of COVID‐19, researchers quickly recognized the need for research on the consequences of the pandemic for agricultural and food systems, both in terms of immediate impacts on access to inputs and labor, disruptions in transportation and markets, and the longer‐term implications on crop productivity, income, and livelihoods. Vegetable production and supply chains are particularly vulnerable due to the perishable nature of the products and labor‐intensive production practices. The purpose of this study was to understand the impacts of COVID‐19 on vegetable production in Burkina Faso in terms of both the biophysical aspects such as yields and access to inputs and socioeconomic aspects such as access to labor, markets, and social services. A survey was developed to better understand smallholder farmer experiences regarding the impacts of COVID‐19 on their vegetable production systems and social well‐being. The survey was administered (between August and October 2020) with smallholder farmers (*n* = 605) in 13 administrative regions covering all agroecological zones of Burkina Faso. The survey results clearly show impacts of COVID‐19 on vegetable systems, including a reduction in access to inputs, a reduction in yields, a loss of income, reduced access to local and urban markets, reduced access to transportation, and an increase in post‐harvest loss. Market access, distribution, and disruptions were a major shock to the system. Results also showed an increase in women's labor in the household, and for youth, an increase in unemployment, job loss, and concerns of poverty. Finally, food security and social supports were highlighted as major issues for resilience and livelihoods. The results from this survey should be helpful to policymakers and researchers to develop policies and strategies to minimize the negative impacts of this ongoing pandemic on the agri‐food systems and support smallholder farmers to overcome stress caused by COVID‐19.

## Graphical abstract

Impacts of COVID‐19 experienced by smallholder vegetable farmers in Burkina Faso were collected through surveys. They expressed reduction in access to inputs, reduced yields, loss of income, reduced access to local and urban markets, reduced access to transportation, and increased post‐harvest loss. These impacted lives, livelihoods, and social well‐being of families, especially women and youth.

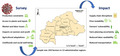

## 1 INTRODUCTION

The multidimensional nature of sustainable food security includes availability, accessibility, nutritional utility, and the stability over time of each of these dimensions (World Food Summit, 1996). Agri‐food systems in West Africa repeatedly reel under abiotic and biotic stresses, poverty, lack of institutional capacity, political conflicts, price volatility, and remain a focus of global food security and attaining the sustainable development goals (SDGs) (Otekunrin et al., 2019). With the increasing emphases on nutritional security and human health in the last few decades, the debate on food security has shifted from an emphasis on a high caloric diet based on grains and tubers to a more varied diet, comprised of energy, vitamin rich, and micronutrient dense foods (Schreinemachers et al., 2018). This drives short‐ and long‐term policy planning for many food programs run by governments, non‐governmental organizations, private entities, and philanthropies around the world. The policy frameworks designed to improve livelihood options and strengthen food security among smallholder farmers in these regions often fail due to inherent uncertainties in planning (Ericksen et al., 2009) and complexities in socioeconomic and biophysical environments (Frelat et al., 2016; Jayne et al., 2014). The agri‐food systems and policy programs have been affected by supply chain disruptions in the COVID‐19 pandemic and now face the challenge of rethinking planning around food and nutritional security to include the more comprehensive notion of sustained livelihood options (Moseley & Battersby, 2020).

Vegetable production is a prime source of micronutrients which complement energy based cereal staples and enhance the household income of growers in Burkina Faso (Schreinemachers et al., 2018) and contributes to both food and nutritional security. Lack of dietary diversity in rural, peri‐urban, and urban regions in Burkina Faso has direct implications for SDGs, some of which revolve around malnutrition, child mortality, mental health, and poverty. Since the 1990s in Burkina Faso, the horticultural sector (which includes vegetables, fruits, and flowers) has emerged as a source of significant agricultural growth and poverty reduction (Hollinger & Staatz, 2015). To cope with chronic food deficits due to prolonged periods of drought and catastrophic flooding—both effects of climate change (Ouédraogo, 2012)—the Government of Burkina Faso has committed itself to developing vegetable crops, which significantly contributes to food security and the fight against unemployment and malnutrition (MAH, 2011; MAHRH, 2007; MARHASA, 2014; MEF, 2010). Vegetable production contributes about 3% of the gross domestic product and was important source of employment in the country (MARHASA, 2014).

In Burkina Faso, vegetable crops are characterized by wide varietal diversity. They are produced in all regions, with variation from one region to another and from one province to another due mainly to the availability of land resources (lowlands in general) and water during the dry season. With the development of water reservoirs and irrigation, vegetable crop production has continued to grow over the years (Knauer et al., 2017). Vegetables are primarily produced in lowlands, around dams, lakes, rivers, reservoirs, streams, and around large urban centers. Vegetable production occurs across the regions; however, there are clear distinctions between urban, peri‐urban, and rural farms (CAPES, 2007). The cultivation of vegetable crops has emerged as a major income generating opportunity for smallholder farmers. The vegetable production system is dominated by onions, tomatoes, cabbage, eggplant, and potatoes and these accounts for about 17% of agricultural production (Kamga et al., 2016; World Bank, 2015).

Since the end of 2019, the rapid transmission and spread of coronavirus disease (COVID‐19) quickly led to a global pandemic. Burkina Faso recorded its first cases in early March 2020. COVID‐19 cases and deaths have slowly risen, with reported confirmed cases of 13,397 and deaths at 164 as of 18 May 2021 (https://coronavirus.jhu.edu/map.html; Worldometer, 2021). Upon the onset of COVID‐19, predictions were made among researchers across the globe concerning impacts of the pandemic on agricultural and food systems that included immediate impacts on access to inputs and labor, disruptions in transportation and markets, and more generally food security and farming systems resilience (Stephens et al., 2020). The longer‐term impact of COVID‐19 disruptions is likely to impact the food systems in the lower income and poor countries with fragile economies and healthcare systems in West Africa (Ali et al., 2020). The disruptions to agricultural value chains caused by COVID‐19 will exacerbate food security challenges in many countries in the sub‐Saharan Africa (Ayanlade & Radeny, 2020) including Burkina Faso (Zidouemba et al., 2020). Our survey on the perceptions of the impacts of COVID‐19 in Senegal indicated that many farmers were concerned and expected negative impacts on their livelihood (Middendorf et al., 2021) due to disruptions in agricultural supply chains and markets. In addition, crop simulation modeling of different scenarios of potential changes in planting areas and yields of major cereal grain crops showed major impacts on total production and its contribution to economies in Senegal and Burkina Faso (Jha et al., 2021). Vegetable supply chains are particularly vulnerable, as these crops are generally perishable and thus at risk of spoilage and post‐harvest loss if there are delays and disruptions along the supply chain, for example in access to labor or timely transportation to markets. Therefore, it will be important to quantify the impact of COVID‐19 on vegetable production systems in Burkina Faso.

The main objective of this research was to study the effect of COVID‐19 on vegetable production in Burkina Faso in terms of both the biophysical aspects such as production and access to inputs, as well as socioeconomic aspects such as access to labor, markets, and social services. We hypothesized that vegetable production would be negatively impacted due to disruptions in inputs supply, access to labor and markets, resulting in increased food insecurity. The results from this survey can help to quantify the impacts of COVID‐19 on vegetable production systems, understand the options taken by producers as part of the response to COVID‐19, and to contemplate strategies to strengthen and build resilience of their farming systems to minimize the impact of such shocks.

## 2 MATERIALS AND METHODS

### 2.1 Study area

**FIGURE 1:**
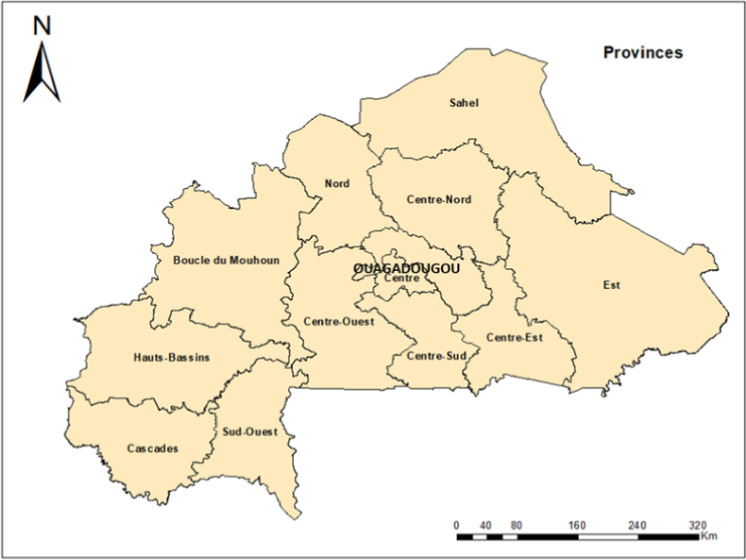
Administrative regions in Burkina Faso where survey was implemented

Burkina Faso is a landlocked country located in the heart of West Africa with an area of approximately 274,200 km^2^, of which 22% (~6 million hectares) is arable land and only 46% of the arable land is currently in use (World Bank, 2021). Burkina Faso's economy is based on the agricultural sector which employs around 80% of the working population and contributes around 25% to country's GDP (World Bank, 2021). In general, agriculture is predominantly subsistence small scale with average landholding of less than 5 ha (Fritz et al., 2015). In terms of administration, the country has 13 regions (e.g., Boucle du Mouhoun, Cascades, Centre, Centre‐Est, Centre‐Nord, Centre‐Ouest, Centre‐Sud, Est, Hauts‐Bassins, Nord, Plateau‐Central, Sahel, and Sud‐Ouest) and 45 provinces (Figure 1).

The country is subdivided into three main climatic zones according to the average annual rainfall: the Sahelian zone in the North (300–600 mm/year), the sub‐Sahelian (or Sudano‐Sahelian) zone in the center (600–900 mm/year), and the north‐Sudanian zone in the south (900–1200 mm/year). The climate has two seasons: (1) the dry season from November to May, characterized by the presence of the harmattan, a hot wind from the Sahara between December and February, and significant heat in March; and (2) the rainy season which extends from June to October.

### 2.2 Sample population and distribution

The survey design and implementation followed the methodology of standard practices in the field (Dillman et al., 2014; Middendorf et al., 2021). The sample frame was drawn in collaboration with Institut de l’Environnement et de Recherches Agricoles (INERA). INERA is the national environment and agricultural research institute in Burkina Faso with access to smallholder farmers throughout the country. INERA’s research primarily focuses on agriculture, as well as microfinance and sanitation (IPA, 2020). The survey population comprises male and female smallholder farmers spread across all 13 administrative regions of Burkina Faso (Figure 1) with the intent to capture perspectives from a national level. In order to cover the diverse agroecological zones and ensure representation across each of the 13 administrative regions and 45 provinces, the researchers stratified the sample and reported results at the national level. Fifty farmers were targeted per region, which produced an overall sample size of 650 farmers. In order to be included in the survey, the potential respondents needed to be at least 18 years of age, engaged in smallholder vegetable production, and head of the household.

The survey was administered with the assistance of INERA scholars and local enumerators, who were familiar with the local languages, vegetable production practices, and the cultural context. The list of farmers was provided by INERA, and the sample included farmers’ names, region, and contact information. The anonymity of the respondents was maintained by ensuring that individual contact information was not linked to the data results. The survey included introductory instructions informing the respondents that participation was voluntary, that the information they shared would not be linked to them or any individual, and that the data would be reported in aggregate form only. Clarification was also provided indicating that they had the option of withdrawing their participation at any time. As part of the consent process, the first question specifically asked whether they wished to participate in the survey, and an affirmative response indicated their consent to participate in the study. Enumerators were given clear instructions and were trained on how to pose the questions and document the response for consistency and accuracy. Enumerators translated the survey into the local language of the respondents. Once the respondent was contacted, consent was ascertained, and the survey was administered via cellphone. The enumerators then entered the participant responses directly into the Qualtrics^©^ survey system.

### 2.3 Survey design and timing

The survey was designed to capture farmer experiences regarding their vegetable production practices and biophysical conditions as well as social well‐being concerns during the COVID‐19 pandemic. Variables of interest include farmers’ experiences related to the impacts of COVID‐19 on their vegetable production practices, access to inputs, ability to plant, yields, markets, labor, gendered division of labor, food security, and community well‐being. The survey also included demographic questions for disaggregation and analysis purposes. A summary of the questionnaire structure in terms of design, questions, sections, and response scales is provided in Table 1. Of the total sample of 650 potential respondents, 605 agreed to participate in the survey, and 45 declined resulting in a 93% response rate. Data collected from the survey were quantified and analyzed using SPSS, a statistical software package.

**TABLE 1:** Summary of the survey design and questions

Section name	Question No.	Question type(s)	Possible responses
Consent	1.2	Willingness to participate	Will participate/will not
Agronomic and Biophysical Aspects of Systems	2.1 – 2.6	Main vegetables grown; production consumed at home; access to inputs; ability to plant, yields	Vegetable choices, %^a^; agreement^b^ scale
Market Issues	3.1 – 3.3	Access to the local/urban markets; issues related to transportation, distributors, harvest loss, sales	Percentage (%)^a^; agreement^b^ scale
Labor Issues	4.1 – 4.6	Access to on‐farm and off‐farm labor; issues related to finances and availability of labor	Agreement^b^ scale; availability scale^c^, (%)^a^ and open‐ended
Impacts for Women	5.1 – 5.2	Perceptions of what might occur for women due to COVID−19	Agreement^b^ scale and open‐ended
Impacts for Youth	5.3 – 5.4	Perceptions of what might occur for youth due to COVID−19	Agreement^b^ scale and open‐ended
Agricultural Adaptations and Mitigation	6.1 – 6.7	Mitigation plans; COVID−19 impact; contingency plans if any	Yes/no and why; open‐ended for impact and contingency plans
Livelihoods and Social Well‐Being	7.1 – 7.5	Access to food, markets, purchases, cost of food, and labor; access to social services, farm credit, subsides, other financial support; challenges due do COVID−19	Agreement^b^ scale; yes/no; and open‐ended
Demographics	8.1 – 8.7	Relationship and activity with farmer organizations; age; gender; district; household size	Yes/no; amount of time; male, female, prefer not to say; age range; open‐ended; household size

^a^ Percent choices were 0%–25%, 26%–50%, 51%–75%, 76%–100%.

^b^ Used a 5‐point scale (1 = Strongly Agree to 5 = Strongly Disagree).

^c^ Used a 5‐point scale (1 = Much less to 5 = Much more).

The survey was launched on 1 August 2020 and was closed on 31 October 2020. The first documented case of COVID‐19 in Burkina Faso was on 9 March 2020. The number of COVID‐19 cases in Burkina Faso at the time of the survey launch was 1143, and the number of COVID‐19 cases at the close of the survey was 2500, thus more than doubling during the survey administration. According to the World Health Organization data, COVID‐19 cases spiked between September and October 2020 and were relatively stable through the beginning of November 2020 with dramatic increases toward the end of the month through February 2021 (https://covid19.who.int/region/afro/country/bf, Accessed: 8 April 2021) and has been stable since. The dramatic increase of COVID‐19 cases during this timeframe is important to note because local experience with COVID‐19 and media coverage of the growing number of positive cases would have been increasing at the time and therefore would have been salient in the public consciousness.

## 3 RESULTS AND DISCUSSION

### 3.1 Respondent demographics

The survey results represent a total of 605 respondents with 597 completing the demographic questions. Table 2 indicates that 20.3% of the respondents were female farmers (*n* = 121), 79.6% were male (*n* = 475), and one respondent preferred not to answer. More than half of the respondents (62.8%) were between the ages of 35–54. Household size ranged from “2” to “more than 20” family members per household, with an average size of thirteen. The variation in household sizes depended on the village and geographic location.

**TABLE 2:** Socio‐demographic characteristics of respondents. Household size was on a 21‐point scale, (1 = “1” to 21 = “More than 20”): Mean = 12.63, Median = 12, Mode = 15

Variable	Category/Description	Frequency (*n* = 597) (%)
Sex	Female	121 (20.3)
	Male	475 (79.6)
Age	18–24	13 (2.1)
	25–34	90 (15.1)
	35–44	206 (34.5)
	45–54	169 (28.3)
	55–64	90 (15.1)
	65–74	27 (4.5)
	75–84 or older	2 (0.4)
Household size	1–5	37 (6.2)
	6–10	200 (33.5)
	11–15	182 (30.5)
	16–20	122 (20.4)
	More than 20	56 (9.4)

Seventy‐nine percent of the respondents indicated having a strong relationship with a farmer organization, and 57% of these considered themselves to *always* be active in that organization. Nineteen percent indicated that they were active *most of the time*, and 12% were active *sometimes*. The following sections describe the respondents’ vegetable production, adaptations, mitigation activities, and issues related to markets, labor, women, and youth. The results also cover farmers’ livelihoods and social well‐being regarding household and community challenges.

### 3.2 Impact on vegetable production

**FIGURE 2:**
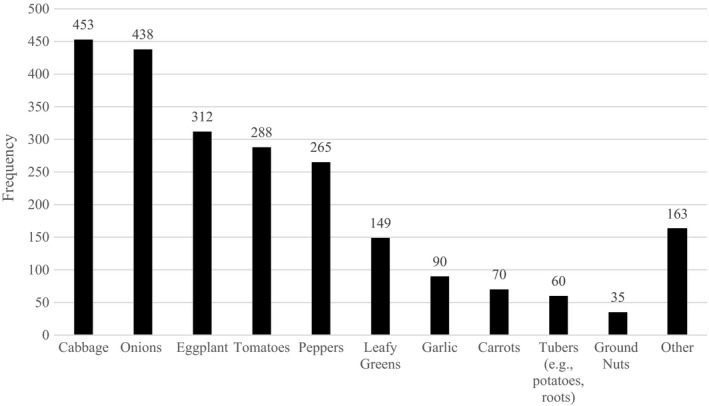
Main vegetables grown among the respondents of the survey (*n* = 604). Note: Other responses described the following “other” vegetables: green beans (*n* = 57), cucumber (*n* = 52), okra (*n* = 27), zucchini (*n* = 19), maize (*n* = 14), rice (*n* = 14), amaranth (*n* = 12), mint (*n* = 5), sorrel (*n* = 4), banana (*n* = 3), papaya (*n* = 3), celery (*n* = 2), percil (*n* = 2), squash (*n* = 2), beterave (*n* = 1), melon (*n* = 1), moringa (*n* = 1), niebe sweet (*n* = 1), pasteque (*n* = 1), rumex (*n* = 1), and strawberries (*n* = 1). If respondents selected “other” but their descriptions reflected other available response options, their responses were recoded to those available response options

Predominant vegetable crops listed by respondents were cabbage (75%), onions (73%), eggplant (52%), tomatoes (48%), and peppers (44%) (Figure 2). According to respondents, additional vegetables grown on their farms included leafy greens (25%), garlic (15%), carrots (12%), potatoes (10%), and groundnuts (6%). Respondents also included a variety of vegetables captured in the “other” category, such as green beans, cucumber, okra, and others.

Additional questions related to production included the understanding how much of the farmers’ vegetable production was consumed at home. According to the survey, 90% of respondents indicated that less than a quarter of their production is consumed at home and the remaining amount was sold at market. This suggests that their vegetable production mostly serves to augment their livelihoods as cash crops, rather than as a primary source of food for their own consumption. Having additional income from vegetable production and/or consumption of vegetables at home are critical to food and nutritional security, especially during a pandemic.

Table 3 highlights farmer responses to what they experienced due to COVID‐19. Specifically, 89.4% of respondents indicated that they experienced a reduction in access to inputs, (e.g., seeds, fertilizers, water, etc.), 51.4% indicated a reduction in their ability to plant vegetables during the planting season, and 84.2% reported a reduction in yields. These percentages are the aggregation of the two response categories “somewhat agree” and “strongly agree.” Based on the 5‐point scale (1 = “strongly disagree” to 5 = “strongly agree”), the means for the responses are “reduced access to inputs” (4.50), “reduced ability to plant vegetables” (3.36), and “reduced yields” (4.14).

**TABLE 3:** Thinking about your experience of what has occurred due to COVID‐19, please indicate your agreement or disagreement with the following statements. COVID‐19 has…

Outcome	Strongly disagree	Somewhat disagree	Neither agree nor disagree	Somewhat agree	Strongly agree	Total	Mean (*SD*)
Reduced my *access* to inputs (e.g., seeds, fertilizers, water, etc.) during this season	25 (4.1%)	20 (3.3%)	19 (3.1%)	107 (17.7%)	434 (71.7%)	605	4.50 (1.00)
Reduced my ability to *plant* vegetables during this season	116 (19.2%)	70 (11.6%)	108 (17.9%)	102 (16.9%)	209 (34.5%)	605	3.36 (1.52)
Reduced my *yields* in the harvest season	42 (6.9%)	30 (5.0%)	24 (4.0%)	214 (35.4%)	295 (48.8%)	605	4.14 (1.16)

Note

Means are on a 5‐point scale (1 = Strongly Disagree to 5 = Strongly Agree).

To further understand the extent of the impact on vegetable production, the survey asked respondents to estimate the percentage of reduction of access to inputs, planted area, and yield. The specific questions read, “how much of a reduction to *access to inputs*, *in the planted area*, and *yield reduction* did you experience due to the impact of COVID‐19?”. The scale options were “none at all,” “0%–25% (less than a quarter),” “26%–50% (less than half),” “51%–75% (more than half),” and “76%–100% (more than three quarters).” Over half (54.6%) of respondents estimated that they experienced *less than half* (40.2%) to *more than half* (14.4%) reduction in access to inputs during the planting season, and 59.8% reported that they experienced a reduction in vegetable yields during the harvest season. The percentages are based on aggregates of the categories of “26%–50% (less than half)” and “51%–75% (more than half).” However, 49.4% of the farmers reported that they did not experience any reduction to their planted area, while the other 48.6% experienced “less than a quarter to less than half” reduction in their planted area. The estimations are based on the aggregates of the categories of “0%–25% (less than a quarter)” and “26%–50% (less than half).” These results suggest that the amount of cultivated land for vegetables did not significantly change, but the access to necessary inputs was drastically reduced and thus farmers experienced a reduction in yields.

### 3.3 Market issues related to COVID‐19 impacts

A major factor in food security is access to markets, especially when the majority of the farmers (90%) reported that they consume less than 25% of their vegetable production at home. Due to the importance of access to rural and urban markets, and issues related to transportation and post‐harvest loss, the researchers inquired about these topics. The findings from the survey clearly illustrate that COVID‐19 had a dramatic impact on farmers’ ability to get their produce to local and urban markets, due to lack of transportation and a reduced number of distributors, resulting in an increase in post‐harvest losses (e.g., spoilage, lack of cold storage, etc.). As shown in Table 4, the majority of respondents experienced reduced access to getting their produce to the local market (94%) and urban market (90%). When aggregating “strongly agree” and “somewhat agree,” 94% of respondents indicated that they experienced a reduction in their ability to transport their produce to the market, and 99% of the respondents agreed that the number of distributors for the produce also was reduced due to COVID‐19.

**TABLE 4:** Thinking about your experience related to *market issues*, please indicate your level of agreement or disagreement with the following statements. COVID‐19 has…

Outcome	Strongly disagree	Somewhat disagree	Neither agree nor disagree	Somewhat agree	Strongly agree	Total	Mean (*SD*)
Reduced access to getting my produce to the *local* market during this season	5 (0.8%)	4 (0.7%)	3 (0.5%)	24 (4.0%)	568 (94.0%)	604	4.90 (0.49)
Reduced access to getting my produce to the *urban* market during this season	5 (0.8%)	–	5 (0.8%)	48 (7.9%)	546 (90.4%)	604	4.87 (0.48)
Reduced my ability to *transport* my produce to the market during this season	9 (1.5%)	3 (0.5%)	25 (4.1%)	59 (9.8%)	508 (84.1%)	604	4.75 (0.69)
Reduced the number of *distributors* for my produce during this season	2 (0.3%)	–	3 (0.5%)	64 (10.6%)	535 (88.6%)	604	4.87 (0.40)
Increased *post*‐*harvest loss* during this season (e.g., spoilage, lack of cold storage, etc.)	5 (0.8%)	2 (0.3%)	2 (0.3%)	72 (11.9%)	523 (86.6%)	604	4.83 (0.52)

Note

Means are on a 5‐point scale (1 = Strongly Disagree to 5 = Strongly Agree).

Lastly, 98.5% of farmers reported having experienced an increase in post‐harvest loss during the season, 44% estimated the amount of loss was more than half (51%–75%) and 35% estimated the loss was between 25% and 50% or less than half. Post‐harvest losses are of particular importance for vegetables due to the perishable nature of the produce. These factors have had a critical impact on food security in Burkina Faso. The salient challenges included difficulties due to market instability, closures, and travel restrictions, which impacted the farmers’ ability to buy and sell produce. The inability to buy or sell produce at the markets had a major impact on farmers’ income and overall food security. One farmer commented “the closures of the markets have shaken up incomes and destroyed living conditions,” and another said “women have had enormous difficulties in feeding their family due to the containment and closure of various markets.”

### 3.4 Labor issues related to COVID‐19 impacts

Another key factor in vegetable production system is access to *on*‐*farm* and *off*‐*farm* labor to support the entire cycle from preparing the land, planting, watering, weeding, harvesting, storage, transport, and marketing. Questions related to finances, ability to hire individuals from within and outside the community, the level of dependence on the source of labor, and challenges faced during the planting and harvesting cycles were asked. As illustrated in Table 5, 73.2% of farmers experienced a reduction of access to labor due to lack of finances during this season, and 57.4% experienced a reduction of access to labor due to a lack of individuals to hire. Meanwhile, 76.6% of respondents reported an increased reliance on household labor during the crop production cycle, which added additional responsibilities and burden for the household unit.

**TABLE 5:** Thinking about your experience of what has occurred due to COVID‐19, please indicate your agreement or disagreement with the following statements related to access to labor. COVID‐19 has…

Statement	Strongly disagree	Somewhat disagree	Neither agree nor disagree	Somewhat agree	Strongly agree	Total	Mean (*SD*)
Reduced access to labor due to a lack of *finances* during this season	39 (6.5%)	30 (5.0%)	92 (15.3%)	51 (8.5%)	389 (64.7%)	601	4.20 (1.24)
Reduced access to labor due to a lack of *individuals to hire* during this season	81 (13.6%)	106 (17.8%)	67 (11.2%)	77 (12.9%)	265 (44.5%)	596	3.57 (1.52)
Increased reliance on *household* labor during this season	104 (17.5%)	22 (3.7%)	13 (2.2%)	129 (21.7%)	326 (54.9%)	594	3.93 (1.51)

Note

Means are on a 5‐point scale (1 = Strongly Disagree to 5 = Strongly Agree).

If the farmer depended on outside labor, they were asked additional questions related to the access to labor throughout the agricultural cycle and the ability to hire workers from within and outside their communities. In response to these questions, 31% of farmers indicated that they do not depend on outside labor. As shown in Table 6, there is somewhat of a mixed response. Almost 40% of respondents indicated “much less” and “somewhat less” access to labor throughout the agricultural cycle; 9.8% reported that it was “about the same” and 19.2% indicated that there was “somewhat more” to “much more” access to labor.

**TABLE 6:** If you depend on outside labor, please indicate the level of access to labor throughout the agricultural cycle

Response option	Frequency	Percent
Much less	70	11.6
Somewhat less	169	28.0
About the same	59	9.8
Somewhat more	80	13.2
Much more	36	6.0
I do not depend on off‐farm labor	190	31.5
Total	604	100

**FIGURE 3:**
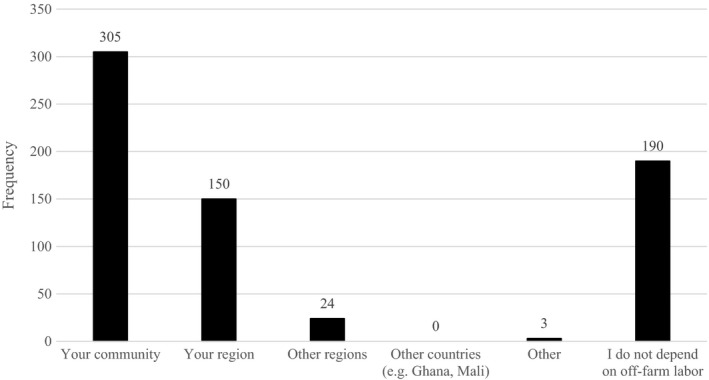
COVID‐19 impact on ability to hire off‐farm labor (*n* = 605)

Results from the ability to hire workers for the production cycle from their community, the region, or from neighboring countries (e.g., Mali, Ghana and Niger), which is very common for Burkina Faso, are also an indication of the hardship faced by farmers due to COVID‐19. As illustrated in Figure 3, 50.4% of respondents were able to hire workers from their community, 24.8% from their region and then their ability to hire labor from other regions or countries drops to 4% from other regions and 0% from contiguous countries.

Based on the qualitative responses from farmers, the inability to hire labor from outside of their communities was attributed to travel and mobility restrictions, health concerns, and other compliance practices dictated by the government. These and other concerns are highlighted in the qualitative analyses from the reported challenges of women, youth, households, and communities, which are discussed later in this article.

The final question related to labor asked whether the farmers were able to hire labor for planting and harvesting during the agricultural cycle. Sixty‐nine percent of the respondents reported that they were able to hire labor, and 31% indicated that they could not hire labor. If the respondent answered “no,” the survey inquired how they were able to handle the situation. Of the 179 respondents who answered this question, 69.2% indicated that their family served as the workforce during the planning and harvesting season. Several farmers indicated that “due to lack of financial means it was my family who helped me plant and harvest.” Farmers also reported that they worked alone (16.8%) and that “their workload doubled more than usual.” Other respondents indicated that they engaged their community members to help with planting and harvesting when possible (7.2%).

### 3.5 Women's issues related to COVID‐19 impacts

The survey specifically explored farmer perceptions of COVID‐19 impacts on women and youth. In Burkina Faso, women play a significant role in agricultural activities (e.g., planting, harvesting, processing) and non‐agricultural activities (e.g., family care, nutrition, marketing). Table 7 illustrates the major impacts of COVID‐19 on women. Respondents (93.9%) indicated that there was a significant increase in women's labor in the household (e.g., meal preparation, water collection, childcare). In terms of women's labor in *on*‐*farm* activities (e.g., planting, weeding, irrigating, harvesting), 75.4% of respondents indicated that there was a significant decrease in these activities due to the increase of household activities and more family members at home due to curfews and travel restrictions. According to the qualitative responses when asked about challenges for women and issues related to labor, the family played a much bigger role in the *on*‐*farm* activities since the farmers did not have sufficient income or financial resources to hire outside the home or family. As an example, one respondent summarized “I had carried out the planting as well as harvesting with my family as I have no money to hire labor.”

**TABLE 7:** Thinking about what occurred for *women* due to COVID‐19, please indicate your one best response to the following statements

Statement	Strongly disagree	Somewhat disagree	Neither agree nor disagree	Somewhat agree	Strongly agree	Total	Mean (*SD*)
There was a significant increase in women's labor in the *household* (e.g., meal preparation, water collection, childcare, etc.)	9 (1.5%)	10 (1.7%)	18 (3.0%)	36 (6.0%)	530 (87.9%)	603	4.77 (0.72)
There was a significant decrease in women's labor in *on*‐*farm* activities (e.g., planting, weeding, irrigating, harvesting, etc.)	33 (5.5%)	80 (13.3%)	35 (5.8%)	169 (28.0%)	286 (47.4%)	603	3.99 (1.25)
There was a significant increase in women's labor in *off*‐*farm* activities (e.g., wage labor, market activities, etc.)	331 (54.9%)	78 (12.9%)	14 (2.3%)	48 (8.0%)	132 (21.9%)	603	2.29 (1.67)

Note

Means are on a 5‐point scale (1 = Strongly Disagree to 5 = Strongly Agree).

When asked whether women experienced a significant increase in *off*‐*farm* activities (e.g., wage labor, market activities), 67.8% of respondents indicated a strong disagreement to the statement. These results also align with the challenges identified for women and their inability to find wage labor off the farm due to massive market closures and travel restrictions. The percentages from the first two statements in Table 7 are the aggregation of the two response categories “somewhat agree” and “strongly agree,” and the percentage for the last statement in the table is the aggregation of the two response categories of “strongly disagree” and “somewhat disagree.” Based on the 5‐point scale, the means for the responses are “increase in women's labor in the household” (M=4.77), “decrease in women's labor in *on*‐*farm* activities” (M=3.99), and “increase in *off*‐*farm* activities” (M=2.29).

The survey also included a qualitative section to further explore the challenges women faced in their household and community due to COVID‐19. Of the 603 responses, the top challenges that women experienced were (1) poverty and financial constraints due to underemployment, market closures, instability, and the lack of ability to buy or sell produce (71%), (2) health concerns and food insecurity in terms of having access to food due to restrictions of travel and confinement (28%), and (3) increase in household chores (10.8%). One respondent commented “women have had enormous difficulties in feeding the family due to the containment and closure of the various markets.” Other challenges faced by women according to respondents were an increase in stress, fear, panic, and sexual harassment.

### 3.6 Youth issues related to COVID‐19 impacts

Respondents were also asked to reflect on how COVID‐19 may have impacted youth labor (see Table 8). When aggregating the response categories of “somewhat disagree” and “strongly disagree,” approximately 50.6% of respondents indicated that they did not experience a significant increase in youth's labor in *on*‐*farm* activities (e.g., planting, weeding) and 86.4% of respondents indicated that there was not a significant increase in *off*‐*farm* activities (e.g., wage labor, market activities). This most likely is due to the extreme loss of jobs and unemployment as well as lack of opportunities outside of the household due to curfews, mobility restrictions, and market closures, as reported by farmers in the qualitative sections of the survey.

**TABLE 8:** Thinking about your perceptions of what occurred for *youth* due to COVID‐19, please indicate your one best response to the following statements

Statement	Strongly disagree	Somewhat disagree	Neither agree nor disagree	Somewhat agree	Strongly agree	Total	Mean (*SD*)
There was a significant increase in local youth's labor with *on*‐*farm* activities (e.g., planting, weeding, irrigating, harvesting, etc.)	216 (35.8%)	89 (14.8%)	12 (2.0%)	42 (7.0%)	244 (40.5%)	603	3.01 (1.81)
There was a significant increase in local youth's labor with *off*‐*farm* activities (e.g., wage labor, market activities, etc.)	363 (60.2%)	158 (26.2%)	16 (2.7%)	23 (3.8%)	43 (7.1%)	603	1.71 (1.16)

Note

Means are on a 5‐point scale (1 = Strongly Disagree to 5 = Strongly Agree).

Farmers were also asked to identify what they considered to be the greatest challenges that were experienced by youth due to COVID‐19, and the vast majority of the responses (94.6%) were related to unemployment, job loss, and concerns of poverty. Other comments related to health concerns, both physical and mental, (10.8%), market instability and closures (10%), reduced access to education (4.5%), and impact from the preventive measures (3.9%) imposed by the government (e.g., restricted mobility, curfews, border closings, etc.). The overall sentiment from many farmers can be expressed by this respondent's comment: “COVID‐19 has destroyed their [youths] jobs and jeopardized their education, and seriously has impacted their well‐being.”

### 3.7 Adaptation and mitigation of COVID‐19 impacts

Smallholder farming systems are complex and require regular interactions assessing tradeoffs and synergies to intentionally determine the best path forward for improved productivity, economic advancement, environmental stewardship, as well as the social and human well‐being of their households and communities (Stewart et al., 2018). Such complex issues are best captured using participatory approaches (Middendorf et al., 2020). Burkina Faso farmers are especially accustomed to assessing tradeoffs and synergies due to the hardships they have faced in their agricultural production and food security. These hardships are primarily due to environmental factors (e.g., drought) and political factors (e.g., terrorist attacks and regional unrest) (Zidouemba et al., 2020). Therefore, researchers wanted to understand what types of agricultural adaptations were undertaken to mitigate the impacts of COVID‐19. The survey posed questions related to changes in vegetable production, traditional agricultural practices, and crop calendars, if any. Table 9 provides a summary of the “yes/no” responses to the questions of whether the farmers changed their vegetable production, traditional agricultural practices, or crop calendars.

**TABLE 9:** When thinking about adaptation and mitigation of COVID‐19 impact

	Frequency	Percent
Changes in types of vegetables you produced		
Yes	43	7.1
No	560	92.9
Changes in traditional agricultural practices		
Yes	9	1.5
No	594	98.5
Changes in crop calendars		
Yes	63	10.4
No	540	89.6
Total	603	100

Note

All three questions were based on (*n* = 603).

The majority of farmers (92.9%, 98.5%, and 89.6%, respectively) did not make any major adaptations or mitigations to combat the impacts from COVID‐19. If the farmer responded “yes” to the above questions, the follow‐up request was to share the changes they made and why. The responses from farmers who indicated that they changed the types of vegetables that they produced (7.1%), the most common change was growing leafy vegetables (e.g., amaranth, okra, and mint), because their production time was relatively short, and they are heat tolerant. Only nine farmers (1.5%) reported a change in their traditional agricultural practices. Some of these changes included cultivating more mint in their nurseries, using herbicides in place of labor, wearing masks, and avoiding groups. Sixty‐three farmers (10.4%) reported that they made changes to their crop calendars due to COVID‐19. The main change was reducing the number of planting cycles during the dry season, which is usually two to three cycles, but due to market closures, poor sales, and lack of space they were only able to plant once.

In the final question related to adaptation and mitigation, farmers were asked what contingency plans they made for their farm, if any. Twenty‐five percent of respondents reported that they did not do anything different and did not have a contingency plan. However, well over half of the farmers (67.8%) reported that their contingency plans involved implementing preventative measures to protect against COVID‐19, such as wearing a mask, washing hands, installing cleaning or sanitation stations on their farms to protect the environment and the community, keeping distance from others, and following the mobility restrictions imposed by the government. The aggregated responses related to agronomic continency plans (5.6%) included increasing organic manure production due to the lack of access to chemical fertilizers, cultivating more cereals (e.g., rice and maize) to compensate for losses in their vegetable production, and seeking other local markets, if possible.

### 3.8 Livelihoods and social well‐being

Table 10 highlights findings regarding perceived COVID‐19 impacts on livelihoods and social well‐being.

**TABLE 10:** Thinking about what occurred due to COVID‐19, please indicate your one best response to the following statements

Statement	Strongly disagree	Somewhat disagree	Neither agree nor disagree	Somewhat agree	Strongly agree	Total	Mean (*SD*)
Getting *enough food* on a regular basis for my household became more difficult	9 (1.5%)	7 (1.2%)	9 (1.5%)	48 (8.0%)	529 (87.9%)	602	4.80 (0.67)
The market where I *purchase* food for my household was either closed or significantly disrupted	6 (1.0%)	3 (0.5%)	3 (0.5%)	46 (7.6%)	544 (90.4%)	602	4.86 (0.53)
There was a significant increase in the *price* of foods that I purchase for my household	6 (1.0%)	10 (1.7%)	30 (5.0%)	82 (13.6%)	474 (78.7%)	602	4.67 (0.73)
The market where I *sell* the produce from my farm was closed or significantly disrupted	5 (0.8%)	3 (0.5%)	8 (1.3%)	69 (11.5%)	517 (85.9%)	602	4.81 (0.56)

Note

Means are on a 5‐point scale (1 = Strongly Disagree to 5 = Strongly Agree).

The majority of respondents reported that it was more difficult to get enough food on a regular basis for their household (95.9%); that the markets where they purchase food was either closed or significantly disrupted (98%); that the price of food increased (92.3%); and the market where they sell their produce was either closed or significantly disrupted (97.4%). These percentages are the aggregation of the two response categories “somewhat agree” and “strongly agree.” Based on the 5‐point scale, the means for the responses are “getting enough food” (*M* = 4.8), “market closure or disruptions (for purchases)” (*M* = 4.86), “increase price of food” (4.67), and “ability to sell produce” (*M* = 4.81).

To augment our understanding of issues related to livelihoods and social well‐being, the survey included questions related to access to social services, farm credit, subsides, and other financial support. Results in Table 11 demonstrate the strong sentiments from farmers with 96.6% indicating disagreement that they had access to social services to help their household, and 98.5% of the respondents disagreed that they had access to farm credit. Similarly, 96.1% of respondents disagreed that they had access to subsidies or other financial supports (98.2%). The results are based on a 5‐point scale where 1 = strongly disagree and 5 = strongly agree. Percentages presented above are the aggregation of the two response categories “somewhat disagree” and “strongly disagree.” Based on the 5‐point scale, the means for the responses are “access to social services” (*M* = 1.23), “access to farm credit” (*M* = 1.16), “access to subsides” (1.24), and “access to other financial supports” (*M* = 1.16).

**TABLE 11:** Thinking about your experiences of what occurred due to COVID‐19, please indicate your level of agreement for each of the following statements

Statement	Strongly disagree	Somewhat disagree	Neither agree nor disagree	Somewhat agree	Strongly agree	Total	Mean (*SD*)
I had access to other *social services* to help my household	509 (84.6%)	72 (12.0%)	7 (1.2%)	2 (0.3%)	12 (2.0%)	602	1.23 (0.68)
I had access to *farm credit*	521 (86.5%)	72 (12.0%)	3 (0.5%)	3 (0.5%)	3 (0.5%)	602	1.16 (0.49)
I had access to *subsidies*	503 (83.6%)	75 (12.5%)	11 (1.8%)	6 (1.0%)	7 (1.2%)	602	1.24 (0.65)
I had access to *other financial support*	523 (86.9%)	68 (11.3%)	6 (1.0%)	2 (0.3%)	3 (0.5%)	602	1.16 (0.49)

Note

Means are on a 5‐point scale (1 = Strongly Disagree to 5 = Strongly Agree).

When asked “what were the greatest challenges that COVID‐19 posed for your household,” 69.4% of the 603 respondents reported issues related to poverty, loss of income, unemployment, and deterioration in living conditions. Other major challenges reported (47.4%) included food insecurity, concerns of health, (both physical and mental), and issues related to preventative measures (e.g., market closures, mobility restrictions, curfews). In the words of one respondent, “COVID‐19 caused us a twist to our family, we can no longer eat as before, we have lost all of our income.”

Similarly, when asked about challenges faced by the community, the main issues were related to poverty, unemployment, and loss of income due to market closures (67.8%). Communities also faced challenges related to implementing the preventive measures and how these restrictions affected their livelihoods (26.7%), increased concerns with health (24.2%), and food security (13.7%). The major health concerns due to COVID‐19 that were reported included the prevalence of panic, fear, anxiety, and stress due to the multiple uncertainties brought on by the pandemic. As one farmer stated: “the biggest challenges that COVID‐19 has posed to the community are health concerns, survival activities, and the ability to make debt payments.”

## 4 CONCLUSIONS

The survey responses clearly showed the impact of COVID‐19 on the vegetable production system and the interconnection between the human health, agri‐food systems, and livelihood in Burkina Faso. The effects on the vegetable production system not only impact the food security and income but also the nutritional security, as vegetables form a key source of nutrition for rural, peri‐urban, and urban populations. Overall, results from our survey point to the devastating impacts from COVID‐19 that smallholder farmers in Burkina Faso have experienced this past year in terms of food insecurity, reduction in labor productivity, limited access to markets, economic disparities, increased concerns around both physical and mental health, and increased hardships for the household and community. These impacts tend to ripple through the food system, affecting production, labor, transportation, markets, incomes, and ultimately livelihoods and social well‐being of the entire household, women, youth, and the community. COVID‐19 was an unexpected and additional shock to an already fragile agri‐food system. These findings shed light on the thematic issue of food system resiliency and its connectivity to the people's food and nutritional security. The results obtained from this survey study will be impactful in framing food policy and subsidies to mitigate the impacts of COVID‐19 on vulnerable segments of society. Moreover, it will also inform end‐to‐end value chain practitioners to develop and proactively adapt their strategies with innovative management of domestic and international trade. There will be clear need for developing country‐ and region‐specific policies, strategies, and reforms after the COVID‐19 pandemic to deliver healthy, nutritious, and safe diets and establish more resilient agri‐food systems that can withstand the sudden shocks either due to climatic conditions, pests and diseases or human pandemics. Other issues revealed by this survey that were beyond the scope of this study warrant further examination such as social well‐being and welfare of women and families concerning physical and mental health, potential inequalities of social and financial services in rural vs. urban locations, and impacts on global trade.

## CONFLICT OF INTEREST

The authors declare that they have neither conflict of interest nor competing interest.
